# Key *auxin response factor* (ARF) genes constraining wheat tillering of mutant *dmc*

**DOI:** 10.7717/peerj.12221

**Published:** 2021-09-21

**Authors:** Junchang Li, Yumei Jiang, Jing Zhang, Yongjing Ni, Zhixin Jiao, Huijuan Li, Ting Wang, Peipei Zhang, Wenlong Guo, Lei Li, Hongjie Liu, Hairong Zhang, Qiaoyun Li, Jishan Niu

**Affiliations:** 1 National Centre of Engineering and Technological Research for Wheat/National Key Laboratory of Wheat and Maize Crop Science, Henan Agricultural University, Zhengzhou, Henan, China; 2 Shangqiu Academy of Agricultural and Forestry Sciences, Shangqiu, Henan, China; 3 College of Life Sciences, Henan Agricultural University, Zhengzhou, Henan, China

**Keywords:** Wheat (*Triticum aestivum* L.), Tillering, Auxin response factor, Expression profiles, IAA

## Abstract

Tillering ability is a key agronomy trait for wheat (*Triticum aestivum* L.) production. Studies on a *dwarf monoculm* wheat mutant (*dmc*) showed that *ARF11* played an important role in tillering of wheat. In this study, a total of 67 ARF family members were identified and clustered to two main classes with four subgroups based on their protein structures. The promoter regions of *T. aestivum ARF* (*TaARF*) genes contain a large number of *cis*-acting elements closely related to plant growth and development, and hormone response. The segmental duplication events occurred commonly and played a major role in the expansion of *TaARFs*. The gene collinearity degrees of the *ARFs* between wheat and other grasses, rice and maize, were significantly high. The evolution distances among *TaARFs* determine their expression profiles, such as homoeologous genes have similar expression profiles, like *TaARF4-3A-1*, *TaARF4-3A-2* and their homoeologous genes. The expression profiles of *TaARFs* in various tissues or organs indicated *TaARF3*, *TaARF4*, *TaARF9* and *TaARF22* and their homoeologous genes played basic roles during wheat development. *TaARF4*, *TaARF9*, *TaARF12*, *TaARF15*, *TaARF17*, *TaARF21*, *TaARF25* and their homoeologous genes probably played basic roles in tiller development. qRT-PCR analyses of 20 representative *TaARF* genes revealed that the abnormal expressions of *TaARF11* and *TaARF14* were major causes constraining the tillering of *dmc*. Indole-3-acetic acid (IAA) contents in *dmc* were significantly less than that in Guomai 301 at key tillering stages. Exogenous IAA application significantly promoted wheat tillering, and affected the transcriptions of *TaARFs*. These data suggested that *TaARFs* as well as IAA signaling were involved in controlling wheat tillering. This study provided valuable clues for functional characterization of ARFs in wheat.

## Introduction

Auxin response factors (ARFs) belong to a subfamily of plant B3 superfamily, and they are a kind of plant-specific transcription factors (Liu & Dong, 2017). A large majority of ARF proteins contain three conserved domains, including a N-terminal B3 DNA binding domain (DBD), a middle region transcriptional activation domain (AD) or repression domain (RD), and a carboxy-terminal Aux/IAA dimerization domain (CTD) (Guilfoyle & Hagen, 2007; Huang et al., 2019).

As whole plant genomic sequences have been reported continuously, *ARF* gene families in many plant species have been systematically analyzed, such as 23 *ARF* genes in *Arabidopsis thaliana* (Okushima et al., 2005), 31 *ARF* genes in maize (*Zea mays* L.) (Xing et al., 2011), 25 *ARF* genes in rice (*Oryza sativa* L.) (Wang et al., 2007), 4 *ARF* genes in millet (*Setaria italica* L.) (Zhao et al., 2016), and 20 *ARF* genes in barley (*Hordeum vulgare* L.) (Huseyin, 2018). These data will significantly promote the functional studies of plant *ARF* genes.

In recent years, a large number of *ARF* genes have been cloned in plants and some of their functions have been studied. *A. thaliana ARF5* (*AtARF5*) is the first plant *ARF* gene isolated by map-based cloning, and it plays an important role in the formation of embryo pattern and vascular tissue (Hardtke & Berleth, 1998). Mutations in *AtARF1* and *AtARF2* affect the growth patterns of pistils, as well as leaf senescence, floral organ abscission (Ellis et al., 2005). *AtARF3* and *AtARF4* play important roles in plant reproductive and nutritional growth (Pekker, Alvarez & Eshed, 2005). *AtARF7* and *AtARF19* promote lateral root formation and play important roles in hormone signaling pathway (Okushima et al., 2005; Feng et al., 2012). Transgenic rice (*Oryza sativa* L.) lines decreasing *O. sativa ARF1* (*OsARF1*) expression are low vigor, stunt growth, have short curled leaves and are sterility, which suggests that *OsARF1* plays an important role in both vegetative and reproductive organ developments (Attia et al., 2019).

Tillering ability is an important agronomic trait for grain production, and tiller bud outgrowth is an important factor determining tiller number (Li et al., 2003). Tiller bud growth is regulated by both genetic and environmental factors, and plant hormones are the direct regulators of both genetic and environmental factors (Zhang & Ma, 2015). The endogenous hormone indole acetic acid (IAA) is indirectly involved in the regulation of tiller bud growth (Choi et al., 2013), IAA is mainly synthesized in the shoot tip and young leaves, and it inhibits tiller bud growth by participating in the apical dominance, thus controlling the tiller occurrence (Ljung, Bhalerao & Sandberg, 2001). ARFs regulate the expression of auxin response genes (Guilfoyle & Hagen, 2007). Current study found that OsmiR167a repressed its targets, *OsARF12*, *OsARF17* and *OsARF25,* to control rice tiller angle by fine-tuning auxin asymmetric distribution in shoots (Li et al., 2020). The transgenic rice plants overexpressing miR167 resulted in a substantial decrease the mRNA amount of four *OsARF* genes, *OsARF6*, *OsARF12*, *OsARF17* and *OsARF25*, remarkably reduced tiller number (Liu et al., 2012).

At present, there are few studies on the regulation of wheat tillering by ARF genes. Research on mutant *dmc* helped us to confirm the importance of miR396b-*TaARF11* in regulating tiller development (He et al., 2018). Besides, subsequent experiments showed that the contents of IAA in Guomai 301 and *dmc* were significantly different (An et al., 2019). In this study, all the ARF family members were identified using the version of wheat reference genome (RefSeq-v1.1) (IWGSC, 2018), and their evolution was studied. We thoroughly investigated the expression profiles of *TaARF* genes in Guomai301 and mutant *dmc* under normal growth and development condition, and exogenous IAA treatment. Also, we measured the endogenous hormone contents and analyzed the correlation between IAA and tiller capacity. These results provided a theoretical base for further research on the functions of ARFs in wheat.

## Materials & Methods

### Plant materials

Guomai 301 is a representative semi-winter wheat cultivar in Henan, China. It has dark green leaves, thick stems, long awns, large spindle-shaped spikes, and an average 37.4 grains per spike. These data were collected as described in previous study (Li et al., 2019).

Mutant *dmc* was obtained from EMS (ethyl methyl sulfonate) treated Guomai 301. The mutant and Guomai 301 were planted in our experimental field. Field management refers to conventional method (Li et al., 2014).

### Tiller sample preparation and transcriptome sequencing

Three bulks of tiller samples were prepared separately at the three - leaf stage (WT1, *dmc* 1; sampling date: November 15th 2018), the over-winter stage (WT2, *dmc* 2; sampling date: January 6th 2019) and the rising to jointing stage (WT3, *dmc* 3; sampling date: February 16th 2019) for RNA extraction and used for qRT-PCR analysis. Wheat tillering had been completed at the rising to jointing stage.

**Figure 1:**
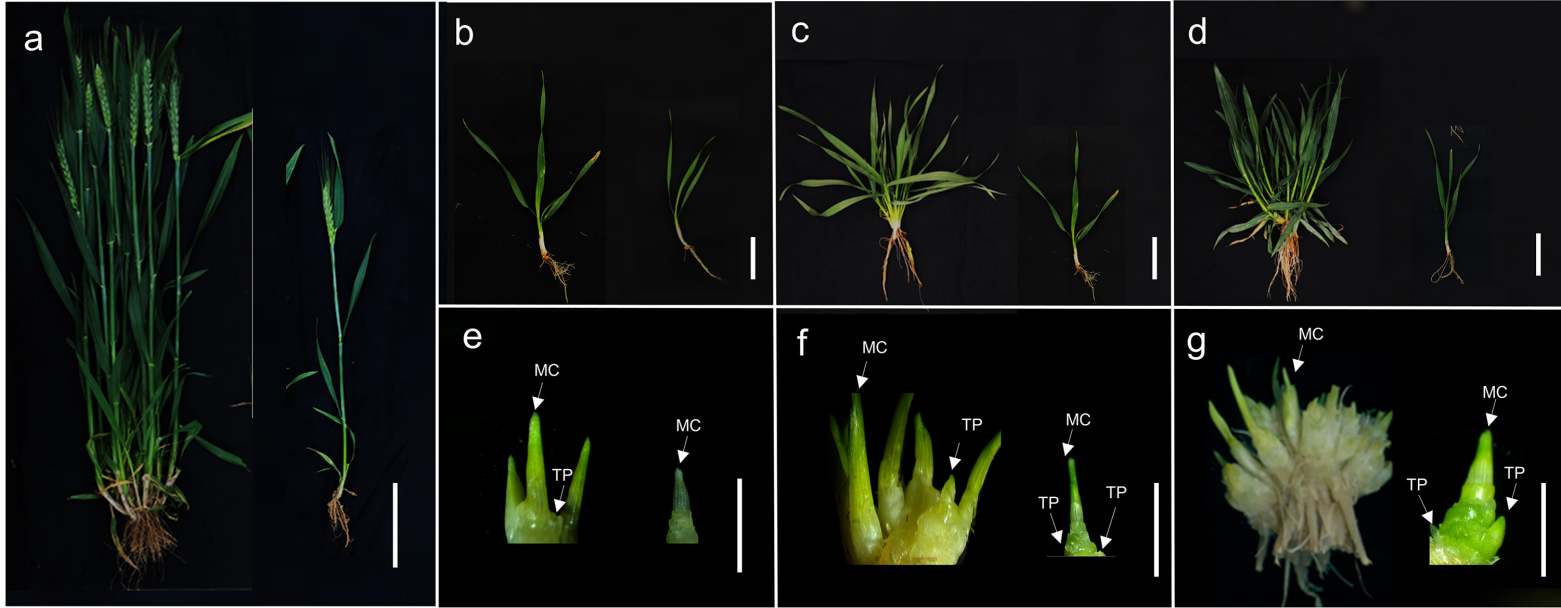
The tiller micromorphology of Guomai 301 (left) and mutant *dmc* (right). (A) The individual plants of Guomai 301 and mutant *dmc* in the field condition. (B) The seedlings of Guomai 301 and *dmc* at the three-leaf stage. (C) The seedlings of Guomai 301 and *dmc* at the over-winter stage; (D) The seedlings of Guomai 301 and *dmc* at the rising to jointing stage. (E) Tiller primordia of Guomai 301 and *dmc* at the three-leaf stage. (F) Tiller primordia of Guomai 301 and *dmc* at the over-winter stage. (G) Tiller primordia of Guomai 301 and *dmc* at the rising to jointing stage. MC: main culm; TP: tiller primordium; Scale bar: 10 cm (A); 2 cm (B–D); 1 cm (E–G).

Tiller primordia of Guomai 301 and mutant *dmc* at the three-leaf stage were dissected to carry out transcriptome sequencing (Fig. 1E). The tiller primordia at the three-leaf stage were carried out RNA-seq. The mutant *dmc* (T01, T02, and T03) and WT (T04, T05, and T06) had three biological replicates, respectively. The transcript abundance of *TaARFs* was calculated as fragments per kilobase of exon model per million mapped reads (FPKM) (Florea, Song & Salzberg, 2013). Differentially expressed genes (DEGs) between two sample pairs were analyzed using the DESeq R package (Wang et al., 2009). The false discovery rate (FDR <0.01) and fold change (FC ≥ 2) were set as the thresholds for DEGs. All analyses were performed on BMKCloud (https://www.biocloud.net/). The bioproject accession of the transcriptome data in NCBI is PRJNA670838. These data were collected as described in previous study (Li et al., 2019).

### Determination of endogenous hormone contents

The tiller samples were prepared separately at the three-leaf stage (T1), the five-leaf stage (T2) and the over-winter stage (T3) for determination of endogenous hormone contents. IAA contents were extracted using a high-performance liquid chromatography method (Fang et al., 1998). Absorbance in each well was measured at 254 nm using a microplate reader (Thermo Scientific C18, Thermofisher, America). The samples at each stage had three independent replicates.

### Continuous treatment of exogenous IAA and the tiller number record

The IAA solution is diluted with distilled water. Data were collected as previously described (Zhang et al., 2021). Specifically, the wheat seedlings of WT and mutant *dmc* at the two-leaf stage were sprayed with 10 µM IAA solution on the leaves until all the leaves were wet, and the controls were sprayed with distilled water. Each seedling was sprayed with 5 mL of water (control) or 10 µM IAA solution. The samples were treated once every three days for a total of 10 times. From the sixth time, the tiller numbers of the plants in different treatments were obviously different. After then, the tiller numbers of the plants were counted every 7 days. The results were analyzed using Excel for Microsoft Office 2016 according to average number.

### Identification and characterization of TaARFs

Data were collected as previously described (Zhang et al., 2021). Specifically, the genome assembly version IWGSC refseqv1.1 (http://plants.ensembl.org/) was used to identify wheat ARF family. Considering that each gene in the wheat genome might have multiple transcripts, amino acid sequence corresponding to the longest transcript was used to identify *ARF* gene. The prediction of ARF proteins from the wheat genome were screened using the Hidden Markov Model (HMM). The HMM files corresponding to the B3 domain (PF02362) and auxin response domain (PF06507) were downloaded from the Pfam database (http://pfam.xfam.org/). HMMER 3.3 (http://www.hmmer.org/) (Finn, Clements & Eddy, 2011) was used to search the *ARF* genes from wheat genome database. All output protein sequences with e-value ≤ 1e−10 were collected. Additionally, keywords ‘ARF’ and ‘auxin response factor’ were employed to search against the Uniprotein database (https://www.uniprot.org/).

After removing all of the redundant sequences, the output putative ARF protein sequences were confirmed by CDD (https://www.ncbi.nlm.nih.gov/Structure/bwrpsb/bwrpsb.cgi), SMART (http://smart.embl-heidelberg.de/) and Pfam (http://pfam.xfam.org/) searching for the presence of the B3 domain and auxin response domain. Finally, obtained *TaARFs* were mainly referred to the annotation information from the Uniprotein database (https://www.uniprot.org/).

### Protein and gene structures, chromosomal locations of ARF genes

The motif distribution was conducted using the MEME online tool (http://meme-suite.org/tools/meme). Parameters were set as following: the motif discovery mode was classic mode, the site distribution was Zero or One Occurrence Per Sequence (zoops), the maximum number of motif finding was 8, and other parameters were default. For exon-intron structure analysis, the DNA and cDNA sequences corresponding to each predicted protein from the wheat genome database were downloaded. The chromosomal map showed the physical locations of all identified *ARF* genes. All images were drawn using TBtools software (Chen et al., 2020). The prediction of isoelectric point (pI) and molecular weight (mw) of *ARF* genes were obtained from the ExPASy Proteomics Server (https://web.expasy.org/compute_pi/).

### Analysis of the cis-acting elements in TaARF promoters

The 2000 bp upstream sequences of transcription start positions of *TaARFs* were extracted to carry out the analysis of *cis*-acting elements. The analysis was completed using the Plant CARE database (http://bioinformatics.psb.ugent.be/webtools/plantcare/html).

### Chromosomal distribution and gene duplication

All *ARF* genes were mapped to wheat chromosomes based on physical locations information from the database of wheat genome using Circos (Krzywinski et al., 2009). Multiple Collinearity Scan toolkit (MCScanX) was adopted to analyze the gene duplication events, with the default parameters (Wang et al., 2012). Non-synonymous (ka) and synonymous (ks) substitution of each duplicated *ARF* gene were calculated using KaKs Calculator 2.0 (Wang et al., 2010). The syntenic maps were drawn using the Multiple Systeny Plot software (https://github.com/CJ-Chen/TBtools).

### Phylogenetic analysis and classification of wheat ARF genes

A total of 23 *ARF* genes in Arabidopsis were obtained from TAIR database (https://www.arabidopsis.org/). A total of 25 *ARF* genes in rice and 31 *ARF* genes in maize were obtained from the Uniprotein database (https://www.uniprot.org/). The phylogenetic trees of the four species’ *ARF* genes were drawn using Neighbor-Joining (NJ) method of MEGA7.0 (http://www.megasoftware.net/), with the following parameters: Poisson model, pairwise deletion, and 1,000 bootstrap replications.

### Analysis of ARF gene expression in various organs or tissues in wheat

The raw gene expression data were downloaded from the Wheat Expression Browser (http://www.wheat-expression.com/). A total of 13 RNA-sequencing data from wheat cultivar Chinese Spring were analyzed. These data were prepared from 13 tissues, including seeding, root, stem, flag leaf, spike, spikelet, awn, glume, lemma, anther, grain, stamen and pistil. Gene expression levels were estimated by the transcripts per million (TPM) values, and presented as log_2_-transformed normalized TPM. The heat map was drawn by TBtools software.

### IAA treatment for gene expression analysis

The seeds of Guomai 301 and *dmc* were set in petri dishes for germination. After three days, the germinated seeds were planted in pot with soil and placed in a growth chamber at 23 °C and 50% relative humidity (RH), the light cycle was 16 h of light and 8 h of dark. The wheat seedlings at the early three-leaf stage were sprayed with distilled water, 1 × 10^−5^ mol/L IAA solution on the leaves, respectively. IAA was diluted with distilled water. The spray was completed until all the leaves were wet.

The tiller primordia of the seedlings sprayed with distilled water were sampled immediately and regarded as a control. The tiller primordia of the seedlings sprayed with IAA solution were sampled at 1 h and 2 h after treatments. All tiller primordia were dissected out with an anatomical needle after the out leaves and sheaths of seedlings were removed. The RNA samples of all treated tissues were immediately extracted and performed subsequent experiments.

### qRT-PCR

Real time qRT-PCR was carried out as described in previous study (Li et al., 2019). Since the homoeoalleles of most tri-genes exhibited similar expression levels (Pfeifer et al., 2014), we used universal primers to analyze the expressions of *TaARF* homoeoallele genes. A total of 20 pairs of primers were designed based on the consensus sequences of homoeoalleles for every wheat *ARF* member, and the primers were listed in Table S1. The *β*-actin gene was used as an internal control and each reaction was performed with three biological replicates. The relative expressions of *TaARFs* were calculated by 2^−ΔΔCT^ methods (Livak & Schmittgen, 2001).

### Statistic analysis

All data were statistically analyzed. Values shown in the form of means ± SD were from three independent experiments. An asterisk (*) and two asterisks (**) indicate significant difference (*P* < 0.05) and highly significant difference (*P* < 0.01) using Student’s *t*-tests, respectively.

## Results

### Typical traits of Guomai 301 and mutant dmc

The mutant *dmc* (Fig. 1A) was mutagenized from wheat cultivar Guomai 301 (Fig. 1A). Mutant *dmc* almost didn’t tiller, and only had a main stem, and the plant height of the mutant *dmc* was significantly lower than that of the WT. At the three-leaf stage (Figs. 1B, 1E), two small tillers grew out at the base of the main culm in WT. Meanwhile, only one tiny protuberance formed at the main culm base of *dmc*. At the over-winter stage (Figs. 1C, 1F), the tiller number of WT was more than 6, while there were only two tiny tiller primordia (TPs) at the base of the *dmc*. Between the rising stage and the jointing stage (Fig. 1D), the tiny TPs of *dmc* were almost unchanged as before (Fig. 1J); but the tiller number of WT had reached its maximum value (Fig. 1G) (An et al., 2019).

### The content change of endogenous IAA during wheat tiller formation

**Figure 2:**
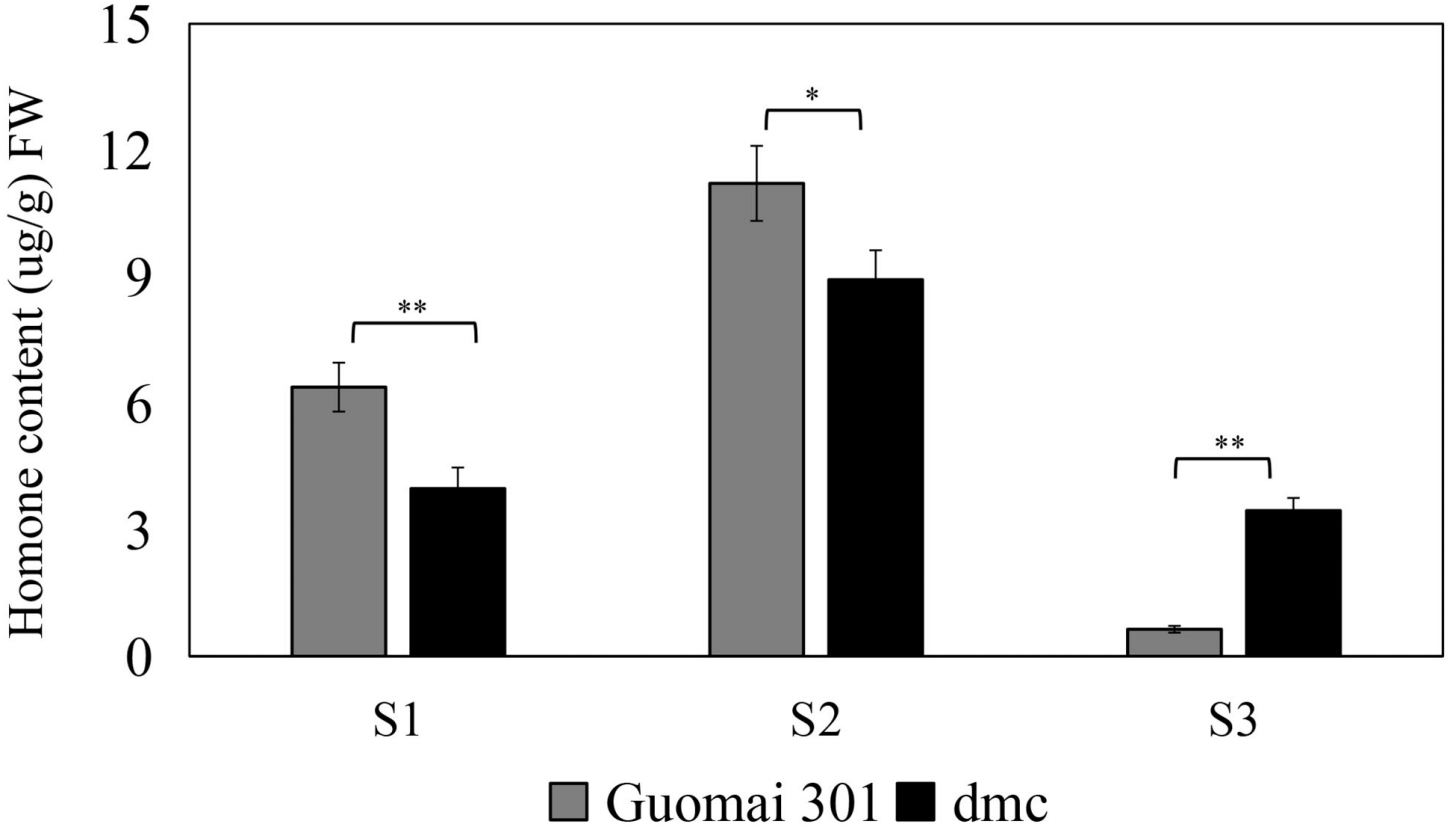
The endogenous IAA contents in tiller primordia of Guomai301 and *dmc*. S1: the three-leaf stage; S2: the five-leaf stage; S3: the over-winter stage. Asterisks indicate significant difference or highly significant difference between Guomai 301 and *dmc* in different stages.

The IAA contents in *dmc* were significantly less than that in Guomai 301 at the three-leaf stage and the five-leaf stage (Fig. 2), and the IAA contents in Guomai 301 were 1.6-fold and 1.3-fold of that in dmc, respectively. While the IAA content in Guomai 301 was significantly less than that in dmc at the over-winter stage, the content of IAA in dmc was 5.4-fold of that in Guomai 301. Besides, the contents of IAA in Guomai 301 and dmc were increased at the five-leaf stage and decreased at the over-winter stage, indicating IAA played essential roles in wheat tiller growth and development.

### Effects of exogenous IAA on wheat tiller formation

**Figure 3:**
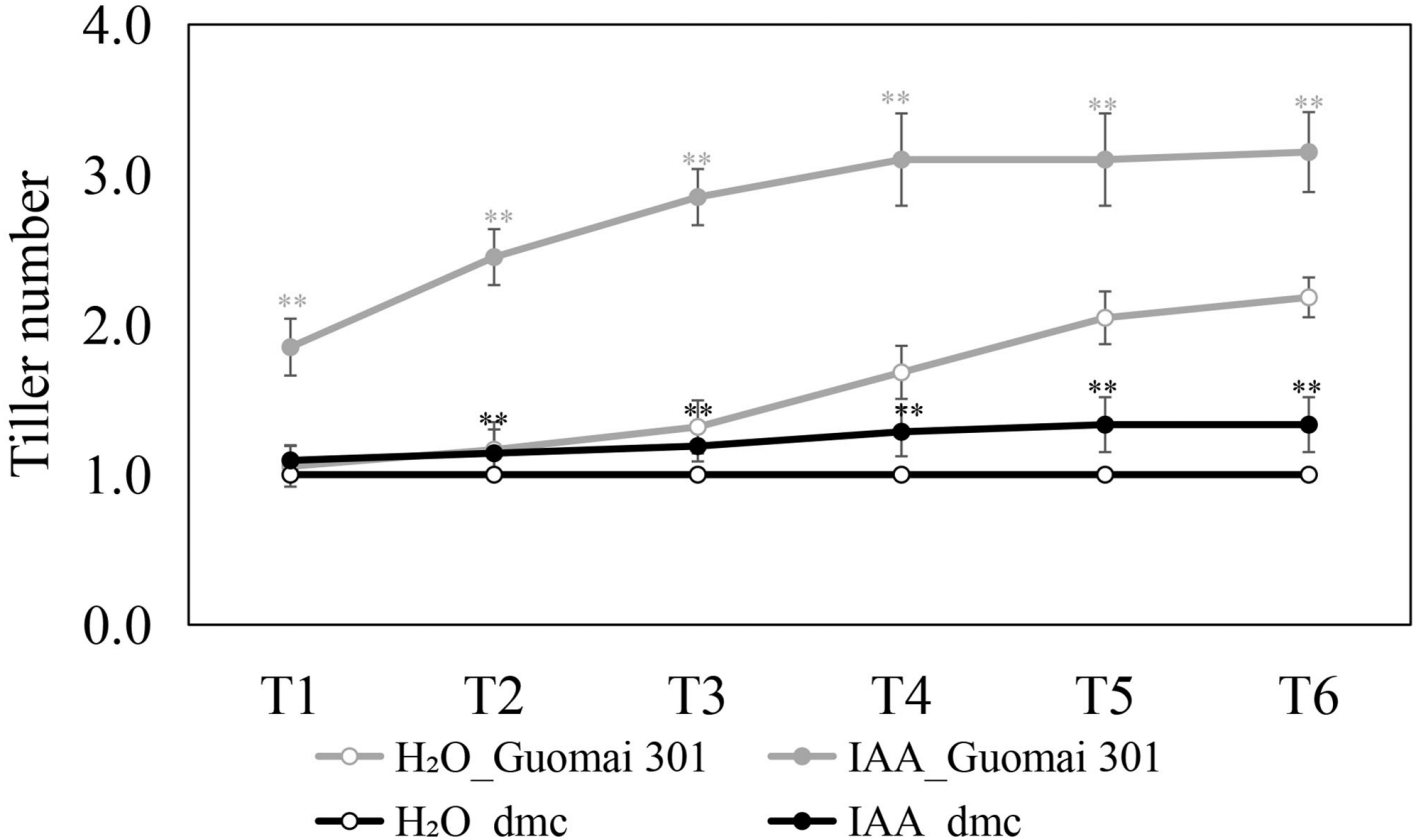
The tiller number changes of Guomai 301 and *dmc* in response to IAA treatments. T1-T6 of the *x*-axis indicated the sampling dates, and the tiller numbers were recorded every 7 days. T1 is the first sampling date which was the 18th day after IAA treatment. Asterisks indicate significant difference or highly significant difference between treated groups and control groups in different sampling dates, respectively.

On the 18th day after IAA treatment (Fig. 3, T1), the tiller number of Guomai 301 was significantly increased, while dmc and the control of Guomai 301 remained no tiller. The exogenous IAA continuously promoted the tillering of Guomai 301, but the effect was less on dmc (Fig. 3). The data indicated that exogenous IAA could significantly promoted tiller development of Guomai 301, but it had less effect on dmc, which suggested that dmc was insensitive to IAA.

### Genome wide discovery of wheat ARFs

A total of 74 candidate ARFs were initially obtained from all wheat protein sequences using HMM (PF02362 and PF06507) by HMMER3.3. The validation of protein conserved domains showed that seven sequences hadn’t AUX_IAA or Auxin_resp domains, which indicated that the seven sequences were not typical ARFs. Eventually, we obtained a total of 67 unique *ARF* genes in wheat. Detailed information about each *ARF* gene was showed in Table S2.

Among the 67 ARF proteins, TaARF13-7D was identified as the smallest protein with 354 amino acids (aa), whereas the largest one was TaARF19-7D with 1175 aa. The molecular weight of the proteins ranged from 38829.72 Da (TaARF13-7D) to 130932.17 Da (TaARF19-7D), and the theoretical pI ranged from 5.42 (TaARF13-2D) to 8.7 (TaARF3-3D).

### Phylogenetic tree of the wheat ARF proteins

**Figure 4:**
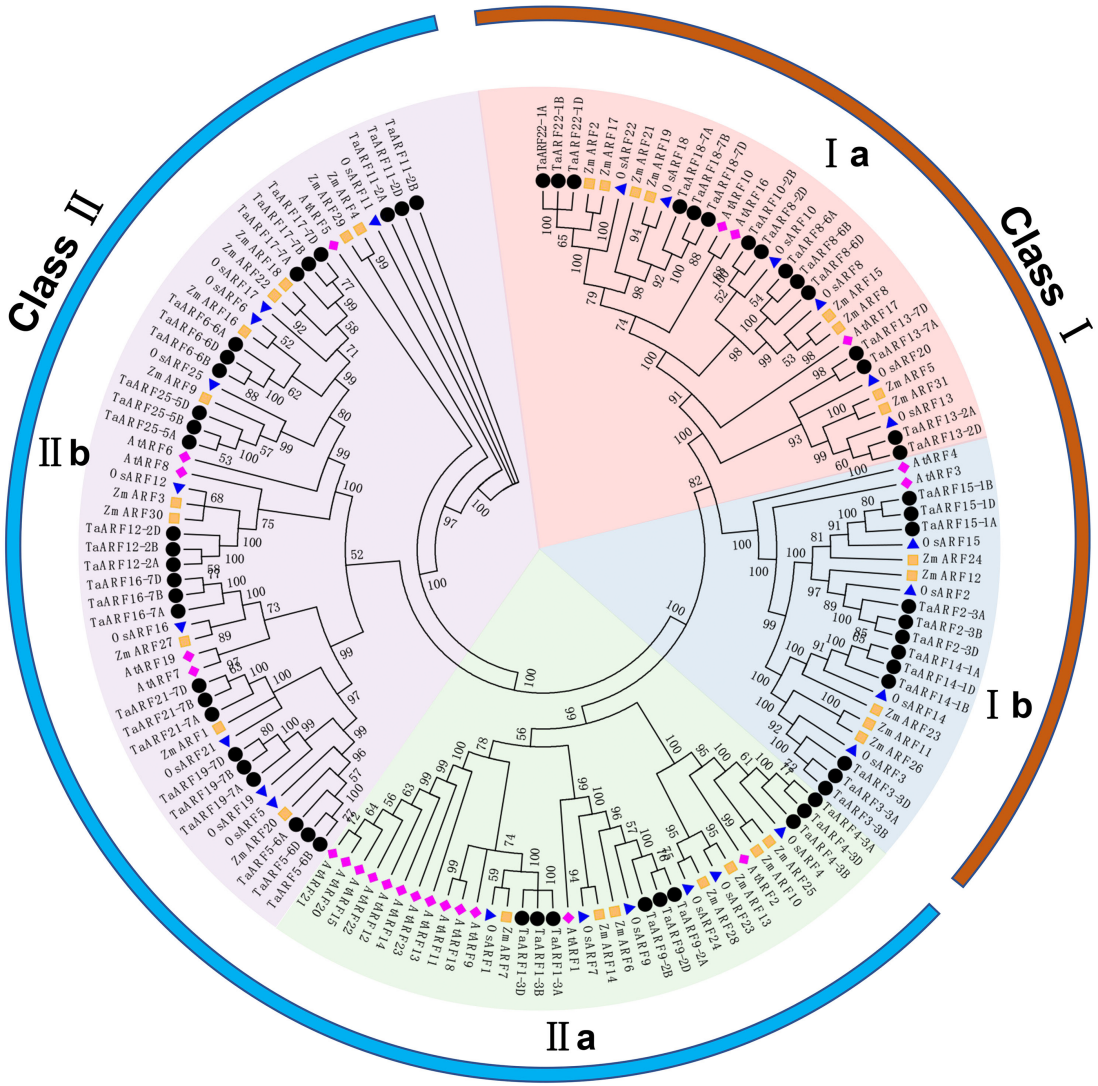
Phylogenetic tree of ARF proteins from Arabidopsis, maize, rice and wheat. The purple solid diamonds represent ARF proteins in Arabidopsis (AtARF); The green squares represent ARF proteins in maize (ZmARF); The blue deltas represent ARF proteins in rice (OsARF); The red solid circles represent ARF proteins in wheat (TaARF); The different colored sectors indicate different groups (or subgroups) of ARF proteins. The different colored arcs indicate different classes of ARF proteins.

An unrooted phylogenetic tree was generated by using the amino acid sequences of a total of 146 ARF proteins from four species (Fig. 4). The result clearly clarified the phylogenetic relationships among the ARFs. According to the bootstrap value of the phylogenetic tree, these ARFs were clustered into two classes (Class I and Class II), including four subfamilies (Ia, Ib, IIa, IIb). Among them, the Class II contained more ARF proteins.

Clustering of protein sequences from different species indicated that the ARFs in the same subfamily were highly similar, which implied their similar functions and evolution processes. Compared to Arabidopsis, wheat ARFs were more closely related to those of maize and rice.

### Motif pattern, domain pattern of wheat ARF proteins

**Figure 5:**
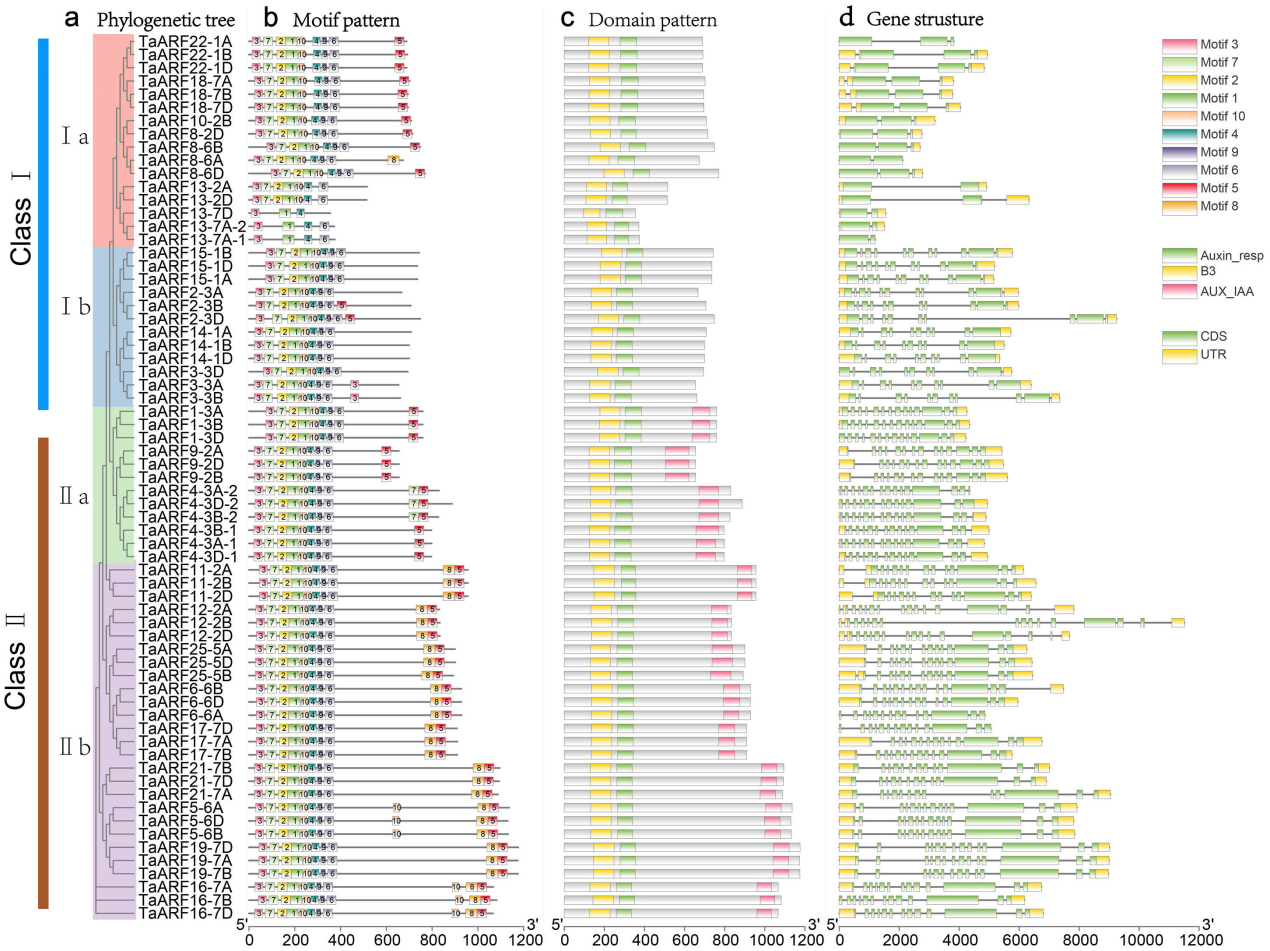
Phylogenetic relationships, conserved protein motif patterns, domain patterns and gene structures of *TaARFs*. (A) The phylogenetic tree of TaARF proteins. Clusters are indicated with different colors. (B) The motif compositions of TaARFs. The 1–10 motifs are displayed in different colored boxes, the scale at the bottom indicates the length of proteins. (C) The domain patterns of TaARFs, the B3 domains are highlighted in yellow, the auxin response domains are highlighted in green, and the AUX_IAA domain are highlighted in lilac. (D) Exon-intron structures of *TaARFs*, yellow boxes indicate 5′- and 3′- untranslated regions; green boxes indicate exons; black lines indicate introns.

To better understand the structural characteristics of ARF proteins in each subfamily, ten conserved motifs were identified in ARF proteins using MEME motif search tool (Fig. 5B, Table S3). Only TaARF13-7D had the least number of motif modules. Motif 1, motif 3 and motif 4 modules were shared by all ARF proteins. Motif 1, motif 2, motif 3, motif 4, motif 5, motif 6, motif 7, motif 9 and motif 10 modules were shared by Class II. Typically, motif 8 existed in subfamily IIb, but without in subfamily IIa.

According to the result of the domain prediction, the proteins in Class I subfamily had B3 and auxin response domains. The proteins in Class II subfamily had B3, auxin response and AUX_IAA domains (Fig. 5C).

### Gene structure of TaARFs

The exon-intron organizations of all the identified *TaARFs* were visualized (Fig. 5D). *TaARFs* possessed two to fourteen exons. Genes within the same group usually had similar structures. For example, all *ARF* genes in Class II contained thirteen exons and fourteen introns, and all *ARF* genes in Class Ia contained three exons and two introns. Among them, *TaARF13*-2A, *TaARF13*-2D, *TaARF13*-7A and *TaARF13*-7D had only two exons.

### Cis-acting elements in the promoters of TaARFs

**Figure 6:**
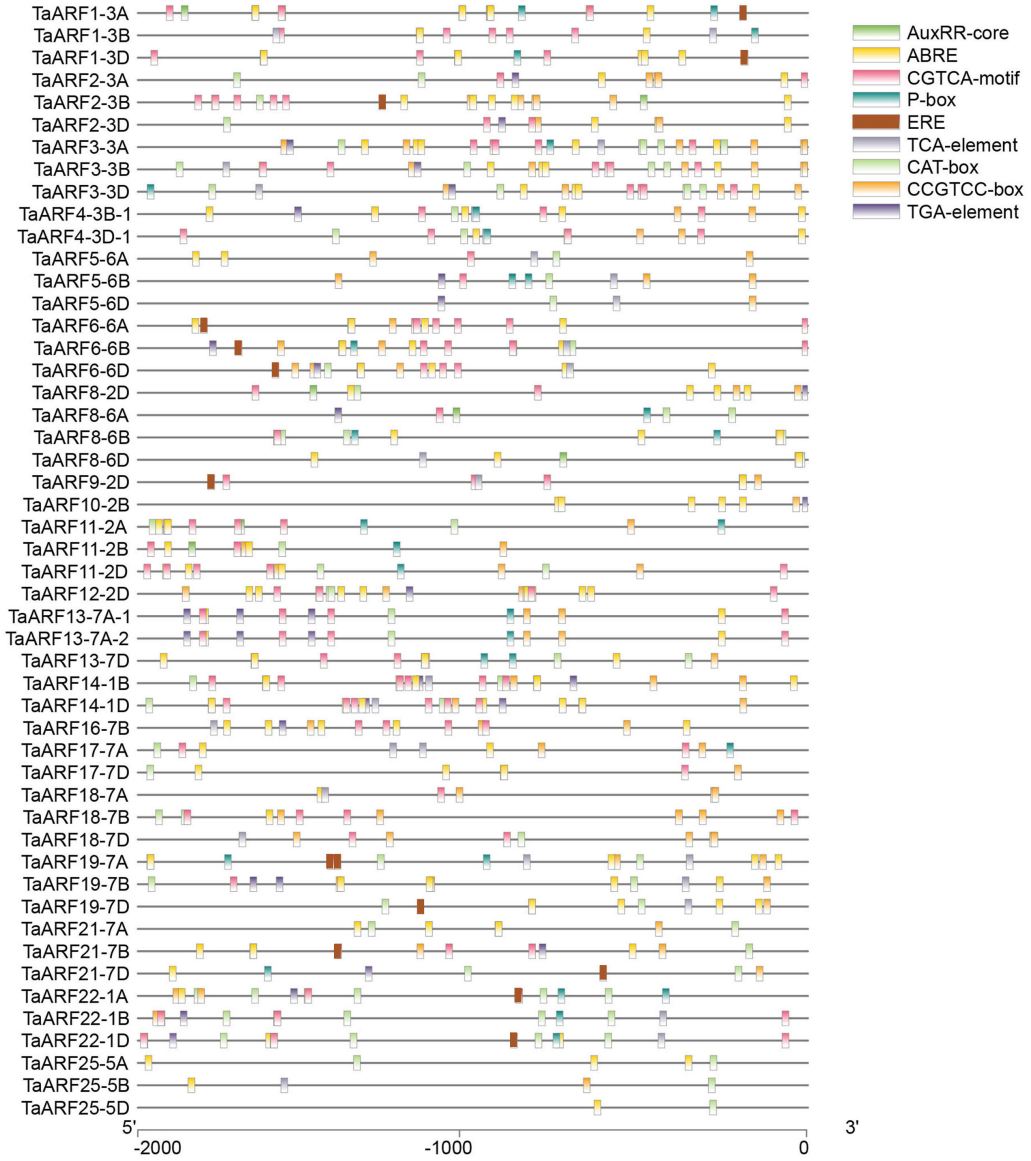
The *cis*-acting elements in the promoters of *TaARFs*. Growth-related *cis*-element: meristem expression regulation (CAT-box and CCGTCC motifs); hormone response-related *cis*-elements: abscisic acid response (ABRE), methyl jasmonate response (CGTCA-motif), salicylic acid response (TCA-element), gibberellic response (P-box), auxin response (TGA-element and AuxRR-core) and ethylene response (ERE).

Among the *TaARFs*, the promoter sequences of 17 *TaARF* genes contained a large number of ‘N’, so they hadn’t been analyzed (Fig. 6). CAT-box and CCGTCC motif *cis*-elements related to growth development exist commonly in the promoter sequences of *TaARFs*. In addition, there are also a large number of hormone response-related *cis*-elements, including some *cis*-acting elements involving in auxin (AuxRR-core, TGA-element), gibberellin (P-box), methyl jasmonate reaction (CGTCA-motif), salicylic acid response (TCA-element), abscisic acid response (ABRE) and ethylene response (ERE). AuxRR-core or TGA-element is the most *cis*-elements, 32 *TaARFs* contain AuxRR-core or TGA-element. Each *TaARF* contains at least two *cis*-elements. For example, *TaARF25-5D* has a growth-related *cis*-element (CAT-box) and a hormone response-related *cis*-element (ABRE). These *cis*-acting elements implied *TaARFs* play various roles in regulating wheat growth and development, and respond to multiple hormones.

### Chromosomal localizations and duplications of TaARF genes

The 67 *TaARFs* were distributed on 18 wheat chromosomes randomly. The majority of *TaARFs* were located on the distal ends of the chromosomes. Chromosome 7A contained the largest number of *ARF* genes (7). No *ARF* gene was identified on the homoeologous chromosomes 4A, 4B and 4D, and only one *ARF* gene and its homoeologous genes (*TaARF25-5A*, *TaARF25-5B* and *TaARF25-5D*) were located on homoeologous chromosomes 5A, 5B and 5D. Four pairs of tandem duplicated genes (*TaARF4-3A-1* and *TaARF4-3A-2*, *TaARF4-3B-1* and *TaARF4-3B-2*, *TaARF4-3D-1* and *TaARF4-3D-2*, *TaARF13-7A-1* and *TaARF13-7A-2*) were located on 3A, 3B, 3D and 7A, respectively. Chromosome 2D, 3A, 3B, 3D and 7A, 7B, 7D had the most *ARF* genes.

**Figure 7:**
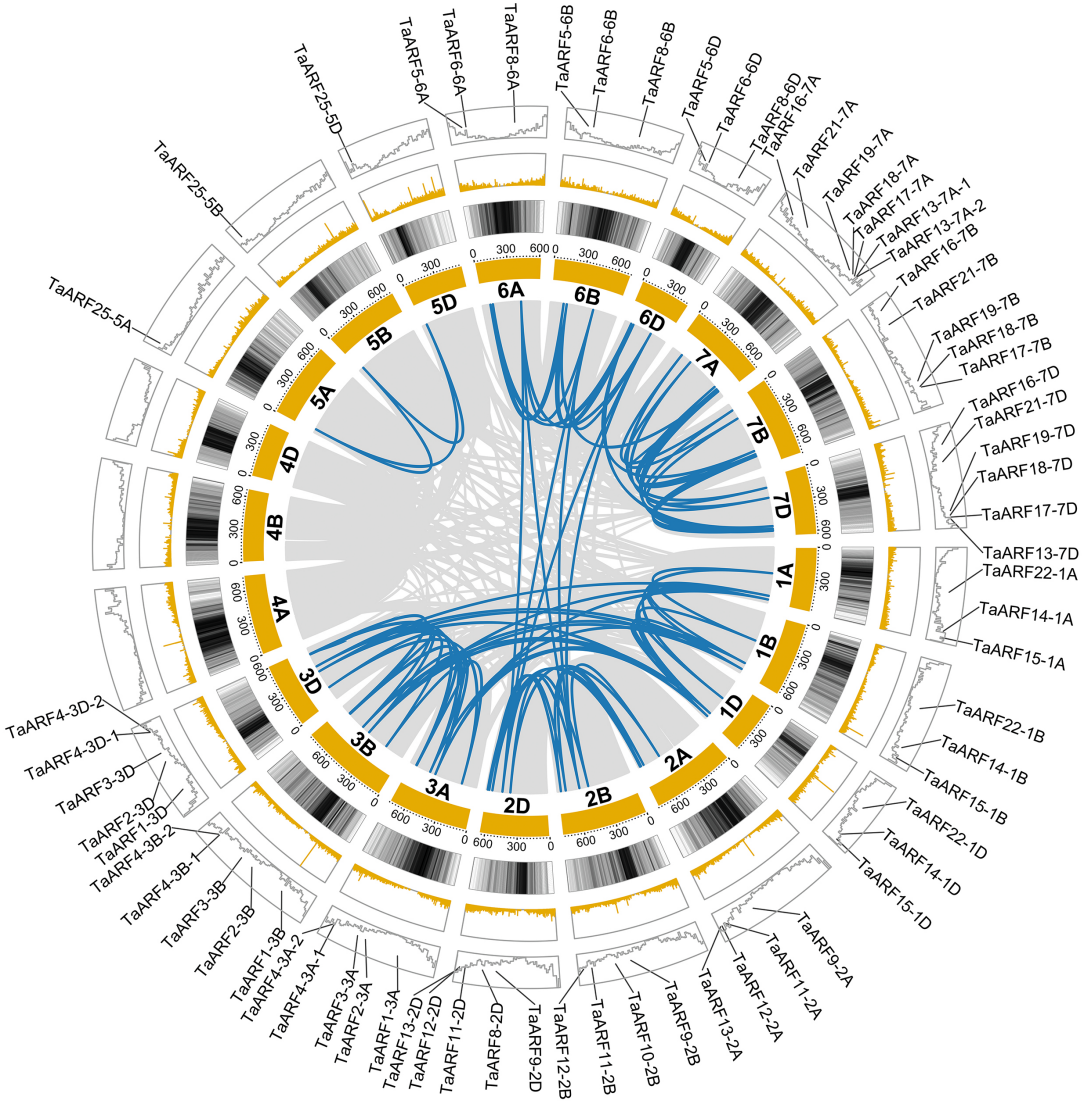
Schematic diagram of the chromosome distribution and interchromosome relationships of *TaARFs*. The grey lines indicate all duplicated gene pairs in wheat, the highlighted red lines indicate probably duplicated *TaARF* gene pairs.

The tandem duplication events (Fig. 7) involving chromosomal localizations of *ARF* genes were used to directly discover the distribution of the duplication of *ARF* genes in the wheat genome. 89 segmental duplication events among 67 *ARF* genes were identified (Table S4). In other words, all *TaARFs* were involved in chromosome segmental duplication. Most *TaARFs* were associated with two to three syntenic gene pairs. Some *TaARFs* had at least three syntenic gene pairs on the same chromosome, such as *TaARF3-3A*, *TaARF3-3B*, *TaARF3-3D*, *TaARF15-1A*, *TaARF15-1B* and *TaARF15-1D*.

Not only chromosome segmental duplication events occurred on the same chromosome, but also occurred between different chromosomes. For example, chromosome 1 and chromosome 3, chromosome 2 and chromosome 6, a total of 19 chromosome segmental duplication events were discovered. These results indicated that the chromosome segmental duplication was a major driving force for *TaARF* evolution.

### Evolutionary relationships of ARF genes in wheat and three different species

**Figure 8:**
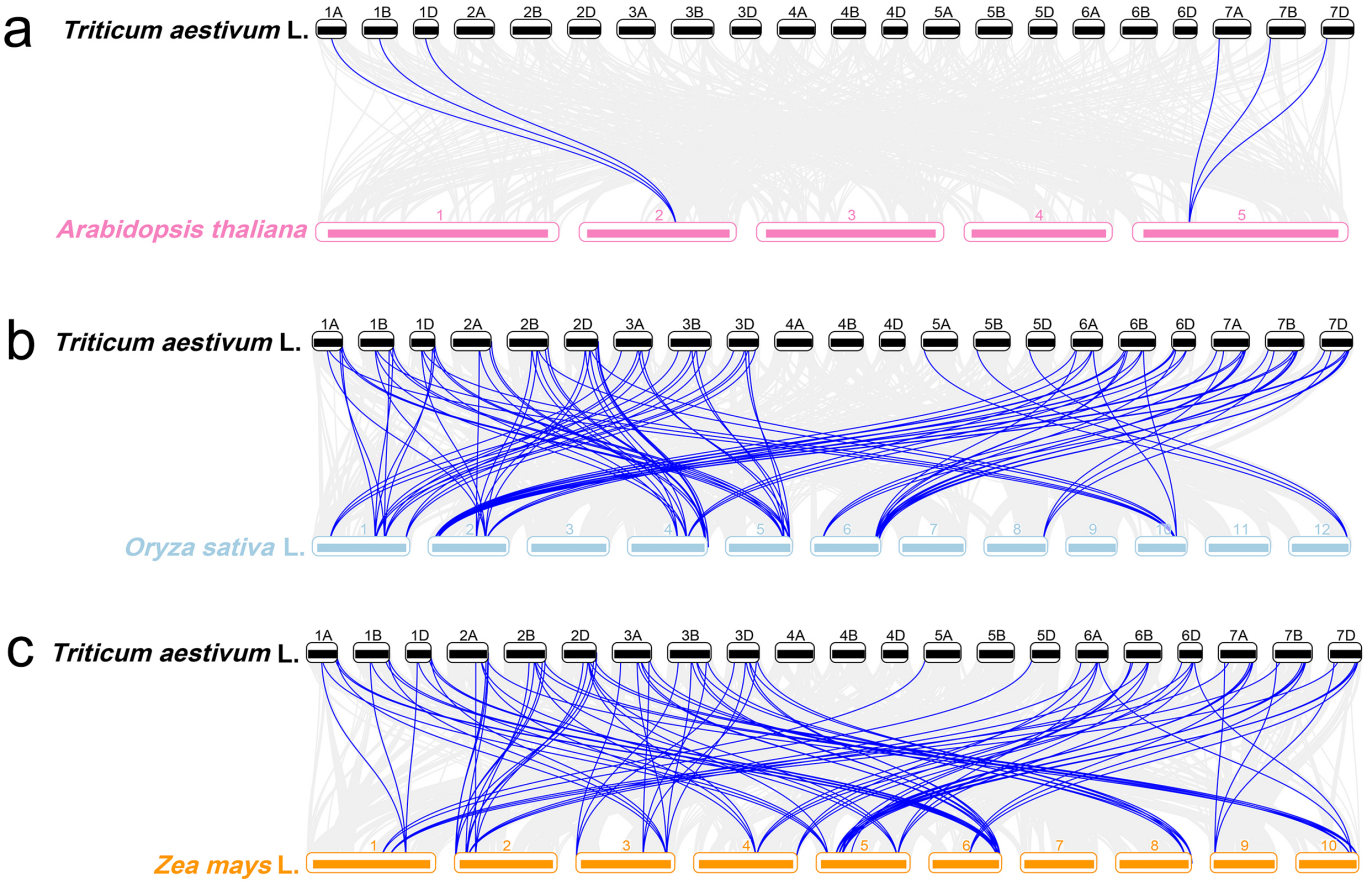
Syntenic relationships of *ARF* genes between wheat and three representative species. Gray lines in the background indicate the collinear blocks within wheat and other plant genomes, while the blue lines highlight the syntenic *ARF* gene pairs.

In order to further understand the evolution mechanism of *ARF* genes among different species. Three comparative syntenic maps associated with wheat genome were constructed with Arabidopsis, rice and maize genomes (Fig. 8). The numbers of the orthologous *ARF* gene pairs between wheat and the three species (Arabidopsis, rice and maize) were 6, 98 and 105, respectively (Table S5).

Six of the 67 *TaARFs* (*TaARF22*-*1A*, *TaARF22-1B*, *TaARF22-1D*, *TaARF16-7A*, *TaARF16-7B* and *TaARF16-7D*) had syntenic relationship with two Arabidopsis *ARF* genes (*AtARF10* and *AtARF7*) (Fig. 8A). *TaARFs* had higher syntenic relationship with grass plants rice and maize (Figs. 8B, 8C). 58 *TaARFs* (including 21 *TaARFs* and their homoeologous genes) had syntenic relationship with 19 maize *ARF* genes (Fig. 8B), 59 *TaARFs* (including 21 *TaARFs* and their homoeologous genes) had syntenic relationship with 22 rice *ARF* genes (Fig. 8C). Especially, the syntenic gene of wheat *TaARF5-6D* was identified in rice, but not in maize.

The Ka/Ks ratios of the *ARF* gene pairs between wheat and other species (Table S5) showed that all segmental and tandem duplicated gene pairs had Ka/Ks <1, suggesting the *TaARF* genes might have experienced strong purifying selective pressure during evolution. In addition, the *ARF* genes in grass plants of wheat, rice and maize were highly conserved in the syntenic blocks, for they had a closer phylogenetic relationship, and these *TaARFs* were evolved from ancient *ARF* orthologous genes.

### The expression patterns of TaARFs in different tissues

**Figure 9:**
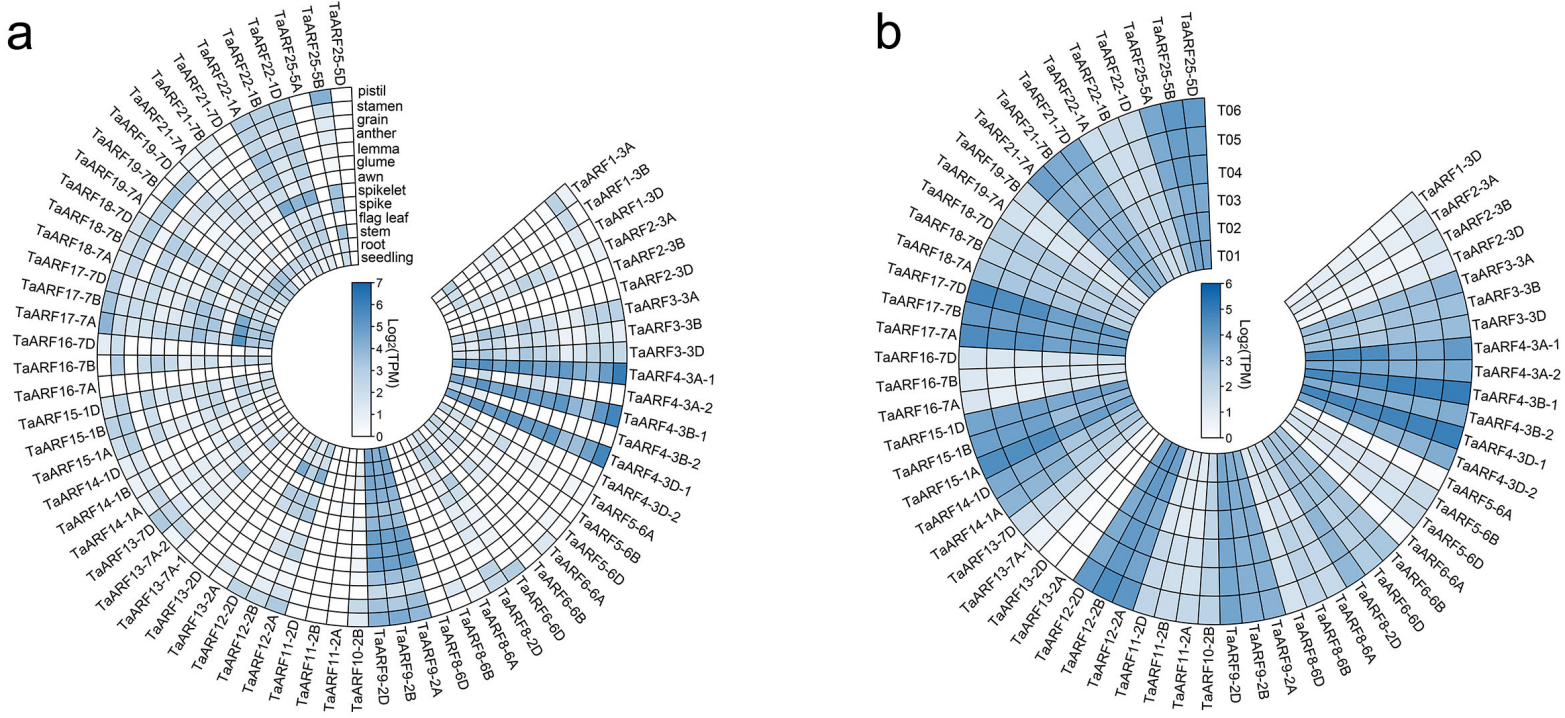
Expression profiles of *TaARFs* in various organs or tissues. (A) Heatmap of expression profiles of *TaARFs* in various organs or tissues of Chinese Spring from the Wheat Expression Browser (http://www.wheat-expression.com/). (B) The heat map of expression profiles of *TaARFs* in tiller primordia of WT and *dmc* based on transcriptome data. Three biological replicates were set up in the mutant *dmc* (T01, T02 and T03) and WT (T04, T05 and T06), and each sample bulk of tiller primordia included more than 10 independent individuals.

The expression profiles of all the 67 *TaARFs* during development were analyzed with the transcriptome data from the Wheat Expression Browser (http://www.wheat-expression.com/), which were derived from 13 wheat organs/tissues at different developmental stages (Fig. 9A). There were four typical expression profiles. (1) *TaARFs* expressed very lowly in all tissues during wheat development, such as *TaARF2*, *TaARF8*, *TaARF11* and *TaARF13*. (2) *TaARFs* expressed highly in all tissues during wheat development, such as *TaARF4* and *TaARF9*. They probably play basic important roles during wheat development. (3) *TaARFs* expressed in all tissues during wheat development, but the expression levels were relative lower, such as *TaARF3* and *TaARF22*. They probably also play basic roles during wheat development. (4) *TaARFs* expressed highly only in specific tissues or their expression levels were changed during wheat development, such as *TaARF17* expressed highly in stem and *TaARF22* expressed highly in spikelet. Most *TaARFs* belong to this class and they play vital roles in various organ developments. Most *TaARF* homoeologous genes had similar expression patterns. Four pairs of tandem duplicated genes (*TaARF4-3A-1* and *TaARF4-3A-2*, *TaARF4-3B-1* and *TaARF4-3B-2*, *TaARF4-3D-1* and *TaARF4-3D-2*, *TaARF13-7A-1* and *TaARF13-7A-2*) showed remarkably different expression profiles, suggesting they evolved from different orthologous genes.

The expression profiles of *TaARFs* in tiller primordia showed that the transcripts of five *TaARF* genes (*TaARF1-3A*, *TaARF1-3B*, *TaARF13-7A-2*, *TaARF14-1B* and *TaARF19-7D*) had not been detected in WT and mutant *dmc*, which indicated their very lower expression levels (Fig. 9B). *TaARF4*, *TaARF9*, *TaARF12*, *TaARF15*, *TaARF17*, *TaARF21*, *TaARF25* and their homoeologous genes had higher expression levels (FPKM>10), but their expressions were not significant differences between WT and mutant *dmc*. High expression levels suggested they played basic important roles during tiller development. In addition, compared to WT, most *TaARF* genes showed low expression levels in mutant *dmc*. Only 4 *TaARF* genes (*TaARF2-3D*, *TaARF11-2A*, *TaARF11-2B* and *TaARF11-2D*) expressed differentially between WT and *dmc* (FC > 2), and they all expressed lowly in mutant *dmc*. Most *TaARFs* expressed relatively lower at early tillering stage in mutant *dmc*, this should be a major factor constraining tillering of the *dmc*.

In summary, *TaARF3*, *TaARF4*, *TaARF9* and *TaARF22* and their homoeologous genes played basic roles during wheat development. *TaARF4*, *TaARF9*, *TaARF12*, *TaARF15*, *TaARF17*, *TaARF21*, *TaARF25* and their homoeologous genes probably play basic important roles during tiller development.

### Expression profiles of TaARFs in tiller primordia of the mutant dmc

**Figure 10:**
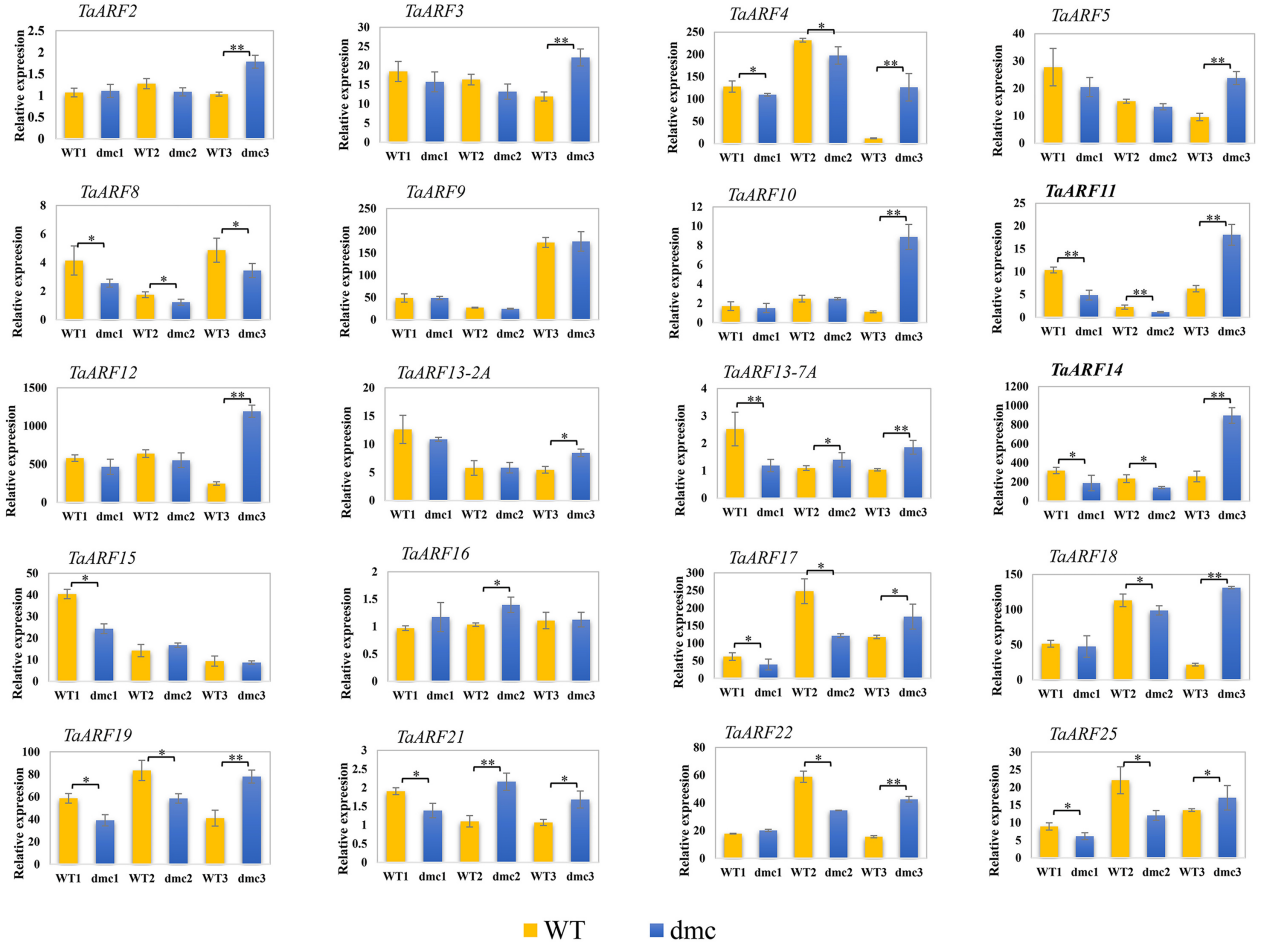
QRT-PCR results of 20 *TaARFs* in the tiller primordia of WT and *dmc* at three tillering stages. WT1, *dmc* 1: the three-leaf stage; WT2, *dmc* 2: the over-winter stage; WT3, *dmc* 3: the rising to jointing stage. Data were normalized to *β*-actin gene and vertical bars indicated standard deviation. Asterisks indicate significant difference or highly significant difference between Guomai 301 and *dmc*.

According to the transcriptomics data, most *TaARF* genes showed no significant differential expressions (FC < 2) at the three-leaf stage. qRT-PCR was performed to analyze the expression patterns of 20 *TaARFs* in the tiller primordia of WT and mutant *dmc* at three tiller developmental stages (Fig. 10), and the samples at the three-leaf stage (WT1 and *dmc* 1) were consistent with the samples of RNA-sequencing. The 20 *TaARF* genes had various expression patterns at three tillering stages.

Among them, *TaARF2*, *TaARF3*, *TaARF13-2A, TaARF16* and *TaARF19* showed no significant differential expressions at three tillering stages, and most *TaARF* genes showed no significant differential expressions at the over-winter stage, except for *TaARF11* and *TaARF17*. At the rising to jointing stage, *TaARF4*, *TaARF5*, *TaARF10*, *TaARF11*, *TaARF12*, *TaARF14*, *TaARF18* and *TaARF22* had higher expression levels in mutant *dmc*. A total of 4 *TaARF* genes showed significant differential expression levels between WT and *dmc* at the three-leaf stage, including *TaARF11*, *TaARF13*-7A, *TaARF14* and *TaARF17*. More importantly, these 4 *TaARF* genes were all down-regulated in mutant *dmc*. It indicated that only a few key genes exerted a significant effect on tiller formation at three leaf stage, the constrained tillering of the *dmc* was associated with the lower expression levels of *TaARFs.* Besides, *TaARF11* and *TaARF14* had similar expression patterns, and they expressed lowly in *dmc* at the over-winter stage but expressed highly at the rising to jointing stage.

In summary, the expression patterns of *TaARF* genes were complex. The abnormal expressions of *TaARF11* and *TaARF14* were major causes in constraining the tillering of *dmc*.

### Expression patterns of TaARFs **in response to IAA**

**Figure 11:**
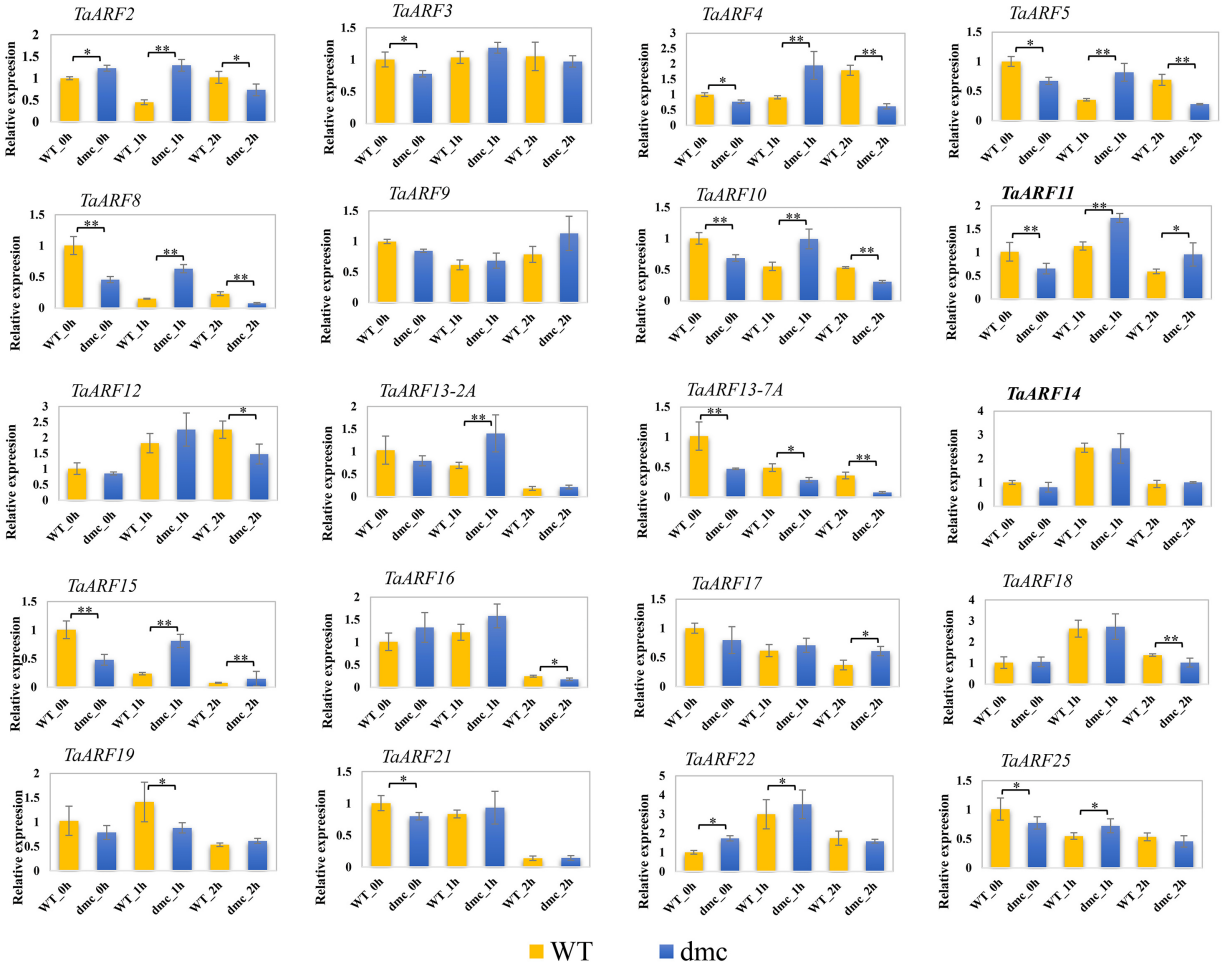
Expression profiles of 20 *TaARFs* in response to IAA treatment. Data were normalized to *β*-actin gene and vertical bars indicated standard deviation. Asterisks indicate significant difference or highly significant difference between Guomai 301 and *dmc*.

The *cis*-acting element analysis showed that a number of hormone response-related *cis*-elements existed in the promoter regions of *TaARF* genes. Typically, *cis*-acting elements involved in auxin regulation. 20 *TaARF* genes were investigated whether their expressions were affected by IAA treatment (Fig. 11).

The expressions of 6 *TaARF* genes (*TaARF2*, *TaARF4*, *TaARF5*, *TaARF8*, *TaARF13-2A* and *TaARF15*) were significantly up-regulated in mutant *dmc* at 1 h after IAA treatment, among the six *TaARF* genes, three *TaARF* genes (*TaARF4*, *TaARF5* and *TaARF8*) were significantly down-regulated in mutant *dmc* at 2 h after IAA treatment. Compare to mutant *dmc*, the expression levels of 7 *TaARFs* (*TaARF9*, *TaARF11*, *TaARF13-2A*, *TaARF15*, *TaARF17* and *TaARF21*) in WT were continuously repressed by IAA treatment, especially, the expression levels of *TaARF15* and *TaARF13-7A* decreased by more than 50% at 1 h and 2 h after IAA treatment. *TaARF13-7A* had the most TGA-element (3) (Fig. 6). The promoter region of *TaARF15* contained a large number of ‘N’, so it was not analyzed. It was speculated that the auxin-related *cis*-acting elements determined the expressions of *TaARFs* response to IAA stimulating. The expressions of *TaARF3* changed not significantly, which suggested it was not sensitive to IAA stimulation. Contrarily, the expressions of *TaARF8* and *TaARF15* were significantly affected by IAA in WT and *dmc*, which suggested they were sensitive to IAA stimulation and might play key roles in regulating wheat tillering.

## Discussion

### Characteristics and evolution of TaARFs

Up to now, *ARF* gene families have been identified in various species, including wheat. In this study, the use of multiple identification methods at the same time greatly improved the accuracy of the wheat ARF genes. A total of 23 wheat ARF members encoded by 68 homoeoalleles are identified from wheat reference genome version TGACv1 (Qiao et al., 2018), and 61 *TaARF* genes are identified from genome version IWGSC1+ popseq.31 (Sun et al., 2018). In this study, 67 *TaARF* genes, including 21 homoeologous *TaARF* loci, distributed on 18 chromosomes were identified in wheat using the latest version of wheat reference genome (RefSeq-v1.1) (IWGSC, 2018), which was the best version of wheat chromosome scale assembly now. The annotation of each *TaARF* gene was carried out referred to the Uniprotein database (https://www.uniprot.org/). Unified annotation will help these results have a wider applicability to the broader field. All these *TaARFs* were highly conserved, and encoded proteins with typical domains of plant ARFs.

Wheat derives from a grass ancestor structured in seven protochromosomes followed by a paleotetraploidization to reach a 12 chromosomes intermediate and a neohexaploidization (involving subgenomes A, B and D) event that finally shaped the 21 modern chromosomes (Pont et al., 2013). Because wheat is a heterohexaploid plant species, it has more *ARF* genes than Arabidopsis (23) (Okushima et al., 2005), rice (25) (Wang et al., 2007) and maize (31) (Xing et al., 2011). The loss of *ARF* genes on chromosome 4 (4A, 4B and 4D) might result from recombinant or modification of some redundant genes during wheat evolution (Chen, 2007; Otto, 2007). Most *TaARF* genes in the same subfamily have similar exon/intron structures, which provide clues to the evolutionary relationships of *TaARFs* (Hu & Liu, 2011). These data indicate that the *ARF* genes with similar structures have similar evolution histories and functions (Babenko et al., 2004; Roy & Penny, 2007). A large number of *cis*-acting elements related to growth and development and hormones regulation existed in the promoter regions of *TaARF* genes, which implied their various functions. *TaARFs* had a poor collinearity with *ARFs* of Arabidopsis, but had a better collinearity with *ARFs* of rice and maize. All *TaARFs* might have happened segmental duplication, which had played a fundamentally important role in *TaARF* evolution (Zhang, 2000; Leister, 2004).

The protein sequences and gene structures of homoeologous genes *TaARF4-3A-1*, *TaARF4-3B-1*, and *TaARF4-3D-1* were highly similar, and that of homoeologous genes *TaARF4-3A-2*, *TaARF4-3B-2*, and *TaARF4-3D-2* were highly similar (Fig. 4, 5, 6), so we concluded that the two homoeologous genes evolved parallel from wheat species formation. The expression profiles of *TaARF4-3A-1*, *TaARF4-3B-1*, and *TaARF4-3D-1* were similar, but were significantly different from that of *TaARF4-3A-2*, *TaARF4-3B-2*, and *TaARF4-3D-2*, which demonstrated the conclusion (Fig. 9). The protein and promoter sequences, and gene structures of *TaARF13-7A-1* and *TaARF13-7A-2* were almost the same, which indicated they were duplicated genes happened not long before (Fig. 6). Except for *TaARF13-7A-1* and *TaARF13-7A-2* were duplicated genes happened recently, the most *TaARFs* were evolved parallel from wheat species formation.

### Various functions of **TaARFs**

Gene structural similarity determines its functional similarity. Plant *ARF* genes in the same subfamily have similar functions (Fig. 4). For example, disruption and overexpression of *AtARF8* affect hypocotyl elongation and root growth habit (Tian et al., 2004). Transgenic experiments show that the *ARF*8 can promote or inhibit lateral root formation in Arabidopsis (Yang et al., 2006). *AtARF4* plays an important role in the reproductive and nutritional growths (Pekker, Alvarez & Eshed, 2005). Similarly, *TaARF4* determines root length and plant height in wheat (Wang et al., 2019). These results indicated that homologous *ARF* genes from different plant species might have similar functions. Most *TaARF* homoeologous genes in A, B and D genomes exhibited similar spatiotemporal expression profiles (Pfeifer et al., 2014), such as *TaARF* 1/4/9/12/15/17/21/25 and their homoeologous genes (Fig. 9). This data also suggested the homoeologous *TaARFs* had similar functions.

Most *ARF* genes have different tissue-specific expression patterns, suggesting their special functions in different tissue/organ development. For example, *ARF7* and *ARF19* regulate lateral root formation in Arabidopsis (Fukaki, Taniguchi & Tasaka, 2006; Okushima et al., 2007). Transgenic Arabidopsis lines expressing *TaARF15-A.1* promotes the growth of roots and leaves (Qiao et al., 2018). *OsARF19* is pivotal for floral organ development and plant architecture (Zhang et al., 2015). *ARF17* is essential for pollen wall patterning in Arabidopsis by modulating primexine formation at least partially through direct regulation of *CalS5* gene expression (Yang et al., 2013), and the overexpression of *ARF17* in the tapetum and microsporocytes of *5mARF17*/WT plants leads to male sterility (Wang et al., 2017). Overexpression of *AtTTP* affects *ARF17* expression and leads to male sterility in Arabidopsis (Shi et al., 2015). Up to now, most functional studies of *ARF* genes have been carried out in *A. thaliana*. Most *TaARFs* also have typical tissue-specific expression profiles (Fig. 9), which suggests their various functions in wheat development.

### The key TaARFs involved in tiller development

Plant *ARF* genes play an important role in maintaining plant stem apical meristem (Zhao et al., 2010). The enhanced miR167 level in transgenic rice resulted in a substantial decrease in mRNA amounts of the four *OsARF* genes, *OsARF6*, *OsARF12*, *OsARF17* and *OsARF25*, the transgenic rice plants remarkably reduced tiller number (Liu et al., 2012). Recent research suggested OsmiR167a could repress *OsARF12*, *OsARF17* and *OsARF25,* to control rice tiller angle by fine-tuning auxin asymmetric distribution in shoots (Li et al., 2020). Our miRNome and transcriptome integrative analysis about the mutant *dmc* and WT found that the highly expressed tae-miR396b (*T. aestivum* microRNA396b) significantly repressed the expressions of *TaGRF* genes and *TaARF11* in *dmc* during tillering (He et al., 2018). It was predicted that the miR396b/*ARF11* regulatory module played a key role in wheat tiller development. Compared with the WT, the expressions of four *TaARFs*, *TaARF11*, *TaARF13-7A*, *TaARF14* and *TaARF17*, in *dmc* were significantly decreased at early tillering stage, which was positively related to the phenotype of *dmc* (Fig. 10). Most *TaARFs* had different expression patterns in WT and *dmc*, but only those significantly differentially expressed *TaARFs* in tiller primordia were the key tiller development regulators. In this case, *TaARF11* and *TaARF14* were significantly differentially expressed at early tillering stage, indicating their important roles in regulating tiller numbers in wheat.

### IAA affect the expressions of TaARFs and significantly promoted tillering

Hormone responses are fundamental to the development and plastic growth of plants (Chapman & Estelle, 2009). There are a number of evidences that exogenous IAA can obviously influence rice and wheat tillering (Kariali & Mohapatra, 2007; Liu et al., 2011; Assuero et al., 2012; Cai et al., 2013). Apically derived auxin does not enter axillary buds directly in several species, including in Arabidopsis (Booker, Chatfield & Leyser, 2003). Apical auxin can inhibit the growth of small buds, and it has been proposed that its inhibitory effect is mediated by a second messenger (Chatfield et al., 2000). In rice, there are many genes related to tiller number may also be related to various plant hormones, rice *dwarf and low tillering 10* (*OsDLT10*) regulates tiller number by monitoring auxin homeostasis (Wen et al., 2020). The phytohormone auxin is involved in almost all developmental processes in land plants, different *ARF* genes probably contribute to the establishment of multiple unique auxin responses in plant development (Roosjen, Paque & Weijers, 2017). In our study, the *TaARF* genes showed various expression patterns after IAA treatment. There are a large number of *cis*-acting elements related to hormones in *TaARF* promoters, including those related to IAA (AuxRR-core, TGA-element). Tissue specific promoters control gene expression in certain organs or tissues (Li & Chen, 2015). The results of qRT-PCR also confirmed that the expressions of *TaARFs* were significantly affected by IAA treatment (Fig. 11). IAA contents in *dmc* were significantly less than that in Guomai 301 at key tillering stages (Fig. 2), and IAA application significantly promoted wheat tillering (Fig. 3). According to these data, it was considered that *TaARFs* as well as IAA signaling were involved in regulating wheat tiller development.

## Conclusions

A total of 67 *TaARFs* were identified in wheat. *TaARF* genes distribute on 18 wheat chromosomes randomly, and their promoter regions have a large number of *cis*-acting elements related to plant growth and development, and hormone response. The most *TaARFs* evolved parallel from wheat formation, except for *TaARF13-7A-1* and *TaARF13-7A-2* duplicated recently. The homoeologous *TaARFs* are highly similar and also have similar expression profiles. *TaARF3*, *TaARF4*, *TaARF9* and *TaARF22* and their homoeologous genes play basic roles during wheat development. *TaARF4*, *TaARF9*, *TaARF12*, *TaARF15*, *TaARF17*, *TaARF21*, *TaARF25* and their homoeologous genes play basic roles during tiller development. The abnormal expressions of *TaARF11* and *TaARF14* are major causes constraining the tillering of *dmc*. The IAA contents of *dmc* are significantly less than that in WT during key tillering stages. Exogenous IAA significantly affected the expressions of *TaARFs* and promoted wheat tillering, which demonstrated that *TaARFs* and IAA signaling were involved in controlling wheat tillering. This study provided valuable clues for functional characterization of ARFs in wheat.

##  Supplemental Information

10.7717/peerj.12221/supp-1 Supplemental Information 1 DNA sequences of the primers used in this study

10.7717/peerj.12221/supp-2 Supplemental Information 2 The basic information of ARFs in wheat

10.7717/peerj.12221/supp-3 Supplemental Information 3 Conserved motif analysis of wheat ARF proteins

10.7717/peerj.12221/supp-4 Supplemental Information 4 One-to-one orthologous relationships of ARFs between wheat and and other species

10.7717/peerj.12221/supp-5 Supplemental Information 5 The duplication gene pairs of ARFs in wheat genome

10.7717/peerj.12221/supp-6 Supplemental Information 6 Raw data for q-RT-PCR

## Additional Information and Declarations

### Data Availability

The following information was supplied regarding data availability: The sequences are available at NCBI BioProject (PRJNA670838) and the raw measurements are available in the Supplemental File.
